# Impaired Formation and Expression of Goal-Directed and Habitual Control in Parkinson’s Disease

**DOI:** 10.3389/fnagi.2021.734807

**Published:** 2021-10-25

**Authors:** Tao-Mian Mi, Wei Zhang, Martin J. McKeown, Piu Chan

**Affiliations:** ^1^Department of Neurology, Neurobiology and Geriatrics, Xuanwu Hospital of Capital Medical University, Beijing Institute for Brain Disorders, Beijing, China; ^2^National Clinical Research Center for Geriatric Disorders, Beijing, China; ^3^Department of Neurology, The Affiliated Hospital of Xuzhou Medical University, Xuzhou, China; ^4^Pacific Parkinson’s Research Centre, University of British Columbia, Vancouver, BC, Canada; ^5^Division of Neurology, Department of Medicine, University of British Columbia, Vancouver, BC, Canada; ^6^Clinical Center for Parkinson’s Disease, Capital Medical University, Beijing, China; ^7^Key Laboratory for Neurodegenerative Disease of the Ministry of Education, Beijing Key Laboratory for Parkinson’s Disease, Beijing, China

**Keywords:** Parkinson’s disease, habitual control, goal-directed control, instrumental learning, stimulus-response task

## Abstract

Selective depletion of dopaminergic neurotransmission in the caudal sensorimotor striatum, a subdivision implicated in habitual control, is a major pathological feature in Parkinson’s disease (PD). Here, we evaluated the effects of PD on the formation of goal-directed and habitual control during learning, and for the first time investigated the conflict between these two strategies in the expression of acquired learning. Twenty PD patients and 20 healthy individuals participated in a set of tasks designed to assess relative goal-directed versus habitual behavioral control. In the instrumental training phase, participants first learned by trial and error to respond to different pictured stimuli in order to gain rewarding outcomes. Three associations were trained, with standard and congruent associations mediated predominantly by goal-directed action, and incongruent association regulated predominantly by habitual control. In a subsequent “slips-of-action” test, participants were assessed to determine whether they can flexibly adjust their behavior to changes in the desirability of the outcomes. A baseline test was then administered to rule out the possibility of general inhibitory deficit, and a questionnaire was finally adopted to test the explicit knowledge of the relationships between stimuli, responses, and outcomes. Our results showed that during the instrumental training phase, PD patients had impaired learning not only of the standard and congruent associations (mediated by goal-directed system), but also the incongruent association (mediated by habitual control system). In the slips-of-action test, PD patients responded less for valuable outcomes and more often to stimuli that were associated with devalued outcomes, with poor performance predicted by symptom severity. No significant difference was found between PD and healthy subjects for the baseline test and questionnaire performance. These results collectively demonstrate that the formation of both goal-directed and habitual control are impaired in PD patients. Furthermore, PD patients are more prone to slips of action, suggesting PD patients exhibit an impairment in engaging the goal-directed system with a relatively excessive reliance on habitual control in the expression of acquired learning.

## Introduction

Instrumental behavior is an elementary type of learning whereby subjects learn the consequences of actions to achieve desirable goals or to avoid undesirable outcomes to guide motivated behavior (Shanks, 1993). According to a dual-system theory, two dissociable learning processes can be identified in instrumental behavior for selecting behavioral options (de Wit and Dickinson, 2009): outcome-driven, goal-directed action and stimulus-driven, habitual control response. Goal-directed action that establishes a causal relationship between action and outcome, which requires the learning of novel stimulus-response associations, is flexible, but slow. In contrast, habitual control response triggered directly by cues in the environment, which can proceed without conscious voluntary intervention, is fast and automatic, but less intrinsically inflexible (Jahanshahi et al., 2015). When a particular task is repeated many times, a habitual control system is subsequently developed. Adams and colleagues (Adams, 1982) first demonstrated that instrumental behavior loses sensitivity to incentive value after extensive training, suggesting a gradual shift from the goal-directed action to the habitual control response (de Wit et al., 2009a).

Multiple studies have identified spatially segregated regions in the basal ganglia for the control of goal-directed and habitual actions (Redgrave et al., 2010; Jahanshahi et al., 2015). In healthy humans, a diffusion tensor imaging study demonstrated that white matter tract strength between caudate and the ventromedial prefrontal cortex predicts goal-directed action, and tract strength between posterior putamen and premotor cortex predicts habitual behavioral control (de Wit et al., 2012). Several fMRI studies have also implicated increased activation of the putamen during goal-directed action behaviors (Monchi et al., 2006; Watson et al., 2018), with the relative contribution of the caudate nucleus becoming dominant over the putamen with prolonged instrumental training.

Parkinson’s disease (PD) may be an excellent model to investigate instrumental behavior. It is characterized pathologically by progressive loss of the ascending dopaminergic projection in the basal ganglia (Armstrong and Okun, 2020; Marino et al., 2020). Redgrave et al. (2010) have suggested that PD patients may have a major deficit in habitual control, and may therefore be forced into a progressive reliance on the goal-directed action. However, evidence supporting this claim is not coming easily. Several probabilistic learning experiments indicated that PD patients exhibit a habit memory deficit (Frank et al., 2007; Bellebaum et al., 2016; Freedberg et al., 2017). However, a major confound with these instrumental learning studies is that the paradigms cannot dissociate habitual versus goal-directed control (de Wit et al., 2011). de Wit et al. (2009a, b, 2011) therefore proposed an instrumental learning procedure to distinguish these two strategies. They tested patients with mild PD but found that habit formation was not impaired, and unexpectedly, revealed only a disease severity-dependent deficit in goal-directed actions (de Wit et al., 2011). Previously, de Wit and colleagues (de Wit et al., 2011) only tested the formation of goal-directed/habitual control using the instrumental task and outcome devaluation task but did not investigate the expression of acquired learning in PD patients.

Based on inconsistent results in previous studies, we aim to further clarify whether PD patients exhibit goal-directed and/or habitual formation deficits during instrumental learning with a larger sample size. Moreover, for the first time, we further investigated the conflict between the goal-directed and habitual control strategies in the expression of acquired learning using a new approach in PD literature. To this end, here, we first employ an instrumental learning task (de Wit et al., 2011), that has been successfully used on both healthy volunteers (de Wit et al., 2009a; Chen et al., 2017) and various disorders (Gillan et al., 2011; Sjoerds et al., 2013; Luijten et al., 2020), to establish the formation of goal-directed and habitual behavior in PD patients. Then, we further administered a “slips-of-action” procedure to test the expression of acquired learning, in which the goal-directed and habitual control processes would compete for behavioral control.

## Materials and Methods

### Participants

The experiments were performed according to the Declaration of Helsinki and were approved by the Institutional Review Board of Xuanwu Hospital of Capital Medical University. Written informed consent was obtained from all participants prior to the study. Twenty-two patients diagnosed with idiopathic PD were recruited from the Movement Disorders Clinic of the Xuanwu Hospital. Exclusion criteria were: (i) Impaired cognition (Mini-Mental State Examination [MMSE] ≤ 24); (ii) Moderate or severe depression (Hamilton Depression Rating Scale [HAMD] > 17); (iii) Moderate or severe anxiety (Hamilton Anxiety Rating Scale [HAMA] > 17; (iv) history of Deep Brain Stimulation surgery; (v) comorbidities of neurological disease other than PD; and (vi) left-handedness. The data of two patients were excluded from the final analyses: with one because of the failure of understanding the task well; and one because of technical error, leaving 20 subjects in the PD group. In addition, a group of 20 sex-, age-matched healthy volunteers were recruited as healthy controls (HC).

### Clinical Assessments

All PD clinical assessments were conducted in the practically “OFF” state, i.e., at least after a 12-h withdrawal of anti-Parkinson medication. The Movement Disorder Society Unified Parkinson’s Disease Rating Scale motor score (MDS-UPDRS III) and Hoehn and Yahr (H-Y) stage were used to assess disease severity. For all participants, MMSE and Montreal Cognitive Assessment (MoCA) scales were adopted to assess general cognitive function. HAMD and HAMA were measured to assess affective symptoms.

### Instrumental Task Paradigm

**FIGURE 1:**
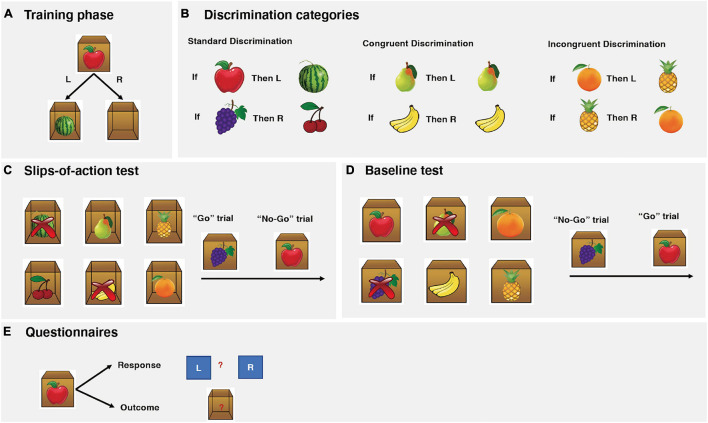
Instrumental task paradigm descriptions. **(A)** The instrumental training phase. In this example from the standard association, participants are presented with an apple stimulus on the outside of the box. If the correct (left) key is pressed, participants are rewarded with a watermelon inside of the box and earned points. If the incorrect (right) key is pressed, an empty box is shown and no points are awarded. **(B)** The three association categories: standard, congruent, and incongruent. **(C)** The slips-of-action test. Six open boxes with outcomes inside are shown, two of which are marked with crosses, indicating these two outcomes are now devalued and will lead to subtraction of points. Subsequently, participants are presented with a series of fruit stimuli. The participants are asked to press the correct keys (“Go”) when the stimulus signals a still-valuable outcome but to refrain from responding (“No-Go”) when the responding outcome has been devalued. In this particular example, participants should press the correct key when the grapes stimulus is depicted (“Go”) but refrain from responding to the apple stimulus (“No-Go”). **(D)** The baseline test. The procedure is identical to the slips-of-action test, except that participants were shown six stimulus fruits (as opposed to outcome fruits). In this particular example, participants should press the correct key when the apple stimulus is depicted (“Go”) but refrain from responding to the grapes stimulus (“No-Go”). **(E)** Questionnaires: Participants were asked to make corresponding response and outcome for each stimulus with paper questionnaires.

The design of the instrumental task paradigm was based on the work of the prior studies (de Wit et al., 2009a; Sjoerds et al., 2013; Chen et al., 2017), which includes the following four tests (Figure 1). For PD patients, the behavioral task was conducted on the same day in the practically “OFF” state as well.

### Instrumental Training Phase

During the training phase, participants learned to respond with a left or right key on a computer keyboard to visually presented stimuli in order to gain outcomes that yield points representing monetary rewards. Participants were presented with a series of closed boxes with pictures of fruits on them (Figure 1A), which appeared on the screen for 2 s (Stimulus). The participants were asked to press either the left or right key (Response) to open the box. A correct response would open the box and reveal another fruit prize inside the box (Outcome) and points were earned. An incorrect response led to an empty box and no points were awarded. The maximum time for each trial was limited to 2 s. All feedback display remained present for 1 s before being replaced by the next stimulus, after a 1.5 s inter-trial interval. During this trial-and-error procedure, the participants learned the correct response for each stimulus and its corresponding outcome. The participants were instructed to make responses as accurately and fast as possible. The faster a response was made; the more points were earned. The number of points awarded for correct responses within the following reaction-time ranges was as follows: 0–1 s, 3; >1–1.5 s, 2; >1.5–2 s, 1. This test consisted of six blocks, with each stimulus appearing twice in random order in each block, resulting in a total of 72 trials.

Three categories of associations were trained together (Figure 1B): standard, cue-outcome congruent, and cue-outcome incongruent. For the standard association, two different kinds of stimulus fruits yielded another two different kinds of fruits as outcomes with correct responses. For congruent association, performing the correct response to a fruit stimulus yielded the same fruit as the outcome. For the incongruent association, each fruit functioned as a stimulus and outcome for opposing responses.

A core feature of the instrumental learning task is the differential involvement of goal-directed and habit learning systems during different categories. Learning the correct response to each stimulus during the standard and congruent association can be established by using either the goal-directed system (Stimulus-Outcome-Response association) or the habitual system (Stimulus-Response association). We would expect behavioral control through direct habitual control to build up concurrently in the habit system (de Wit et al., 2009a). With only limited training performance should, however, be predominantly controlled by the goal-directed system (de Wit et al., 2009a; Gillan et al., 2011; Sjoerds et al., 2013; Chen et al., 2017). However, for the incongruent association, a goal-directed approach is disadvantageous because it leads to response conflict, that is, it causes opposing keys to become associated with the same fruit. Previous studies (de Wit et al., 2007, 2009a, 2011) have demonstrated that both humans and animals tend to rely on a direct Stimulus-Response habitual learning strategy to solve the incongruent association, as opposed to the standard and congruent associations. Therefore, the incongruent association provides a baseline for habitual responses.

### Slips-of-Action Test

Following the instrumental learning phase, a “slips-of-action” test was employed to test the balance between goal-directed and habitual actions. This was accomplished by assessing whether participants were flexible enough to adjust their behavior to changes in the desirability of outcomes. At the start of each block, six different fruit outcomes from the initial training phase appeared on the screen for 5 s (Figure 1C). Two out of the six fruit outcomes were marked with crosses, indicating that these two outcomes were now devalued and the corresponding stimuli would lead to a subtraction of points. Following the instruction screen, a series of closed boxes displaying the fruit stimuli were presented to the participants. If the stimulus was linked with a valuable outcome (“Go” trial), then the correct response should be made; otherwise (“No-Go” trial), any response should be suppressed. Goal-directed action control was thus reflected in the selective responses to valuable as opposed to devalued outcomes. Conversely, if the habitual system was exerting dominant control over behavior, it would result in slips of action toward devalued outcomes. Each closed box was shown for 2 s and was replaced by another box with a different stimulus after a 1 s inter-trial interval. No feedback was given during this test to exclude the possibility of new learning. Participants completed a total of 144 trials over six blocks, with each of the six stimuli shown four times per block in random order. The two devalued fruit outcomes in each block were from different categories and switched across the six blocks, with each fruit outcome being devalued twice throughout the test.

In this test, we also calculated a difference score, by subtracting the percentage of responses to devalued stimuli from the responses to still-valuable stimuli. We then further used a normalized difference score, by dividing the difference score by the percentage of responses to still-valuable stimuli, as a measure of relative goal-directed and habitual control. A higher score would indicate more goal-directed performance (as responses were made to stimuli linked to still-valuable outcomes and appropriately withheld to stimuli linked to devalued outcomes); and conversely, a lower score would indicate more habitual responses that were insensitive to the current outcome value.

### Baseline Test

Subsequently, a baseline test was administered to determine whether the impaired performance in the slips-of-action test was due to a general inhibitory deficit. The procedure was identical to the slips-of-action test, except that the participants were shown six stimulus fruits (as opposed to outcome fruits) from the initial training phase. Outcome retrieval was not required and as such the goal-directed and habitual control didn’t compete for behavioral control for this test (de Wit et al., 2012). This task was therefore used as a control to rule out the possibility that impaired performance on the slips-of-action test was purely related to outcome devaluation insensitivity (Delorme et al., 2016; Ersche et al., 2016).

### Explicit Knowledge Questionnaires

Finally, a questionnaire was assessed to test the participants’ explicit knowledge of the instrumental learning. It consisted of six total questions, each with a response and outcome knowledge component. Participants were asked to indicate whether the right or the left key was correct (“response” knowledge) for each fruit stimulus and which fruit appeared inside the box (“outcome” knowledge) was linked to the fruit stimulus. The questionnaire was scored for each category on the response and outcome, respectively. For example, the subject would score 2 on the response and 2 on the outcome for the standard category, if he/she gave the correct answers that Apple yielded Watermelon with left key, and Grapes yielded Cherries with right key.

### Statistical Analysis

Statistical analysis was performed using Stata 15.1. The normality was tested using Shapiro-Wilk’s test. For the instrumental training test, we conducted repeated-measures ANOVAs on both response accuracy and reaction time, with Category (standard/congruent/incongruent) and Block as within-subject factors, and Group (PD/HC) as between-subject factor. For the slips-of-action and baseline test, we employed repeated-measures ANOVAs on the percentage of responses made, with Devaluation (valuable/devalued) and Category (standard/congruent/incongruent) as within-subject factors, and Group (PD/HC) as between-subjects factor; and performed a two-way ANOVA on the difference scores, with Group (PD/HC) as between-subject factor and Category (standard/congruent/incongruent) as within-subject factor. Bonferroni’s corrections were adopted for multiple pairwise comparisons. *P* < 0.05 was considered statistically significant.

## Results

### Clinical and Demographic Characteristics

Participant demographics and clinical features are shown in Table 1. There were no significant differences in gender, age, education, MMSE, and MoCA scores between the PD and HC groups. Though HAMA and HAMD scores did differ considerably between the two groups, PD subjects had a mean score of 6.50 for HAMA and 7.20 points for HAMD, respectively, which were close to the normal range. However, positive scores could still be expected to affect the instrumental performance. Therefore, we tested the correlation between the response accuracy and HAMA and HAMD scores, respectively, but failed to find any significant correlation (with Pearson correlation of *r* = −0.251, *p* = 0.249 for HAMA score and *r* = −0.072, *p* = 0.745 for HAMD score).

**TABLE 1:** Demographics and clinical features of participants.

Features	PD group (*N* = 20)	HC group (*N* = 20)	T	*p*
Gender (m/f)	11/9	9/11	0.010	0.752
Age	62.15 ± 7.46	59.95 ± 5.31	1.075	0.289
Education (ys)	12.25 ± 3.48	10.75 ± 2.90	1.481	0.147
H-Y stage	2.20 ± 0.68	–	–	–
MDS-UPDRS III	32.35 ± 18.19	–	–	–
Disease duration (ys)	4.95 ± 4.08	–	–	–
LEDD (mg)	518.00 ± 266.26	–	–	–
MMSE	28.20 ± 1.47	28.65 ± 1.35	1.008	0.320
MoCA	24.70 ± 2.45	24.70 ± 2.41	0.000	1.000
HAMA	6.50 ± 3.91	3.35 ± 1.50	3.309	0.002*
HAMD	7.20 ± 4.50	4.45 ± 1.99	2.499	0.017*

Means and SD are shown for continuous variables. H-Y stage, Hoehn and Yahr stage; MDS-UPDRS III, Movement Disorder Society-Unified Parkinson’s Disease Rating Scale motor score; LEDD, levodopa equivalent daily dose; MMSE, Mini-Mental State Examination; MoCA, Montreal Cognitive Assessment; HAMD, Hamilton Depression Rating Scale; HAMA, Hamilton Anxiety Rating Scale HAMA. *p < 0.05.

### Instrumental Training Phase

**FIGURE 2:**
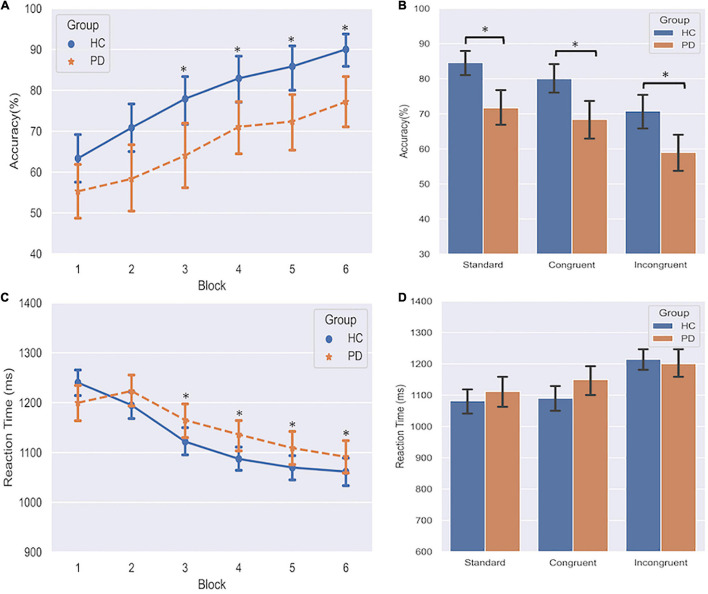
Effects of PD on instrumental learning. **(A)** Response accuracy of PD and HC group over six blocks. *indicates *p* < 0.05 compared to Block 1. **(B)** Response accuracy across acquisition of standard, congruent, and incongruent associations. *indicates *p* < 0.05 in the comparison between PD and HC. **(C)** Reaction time of PD and HC group over six blocks. *indicates *p* < 0.05 compared to Block 1. **(D)** Reaction time across three associations. Error bars denote standard deviations.

The three-way repeated measures ANOVA on response accuracy revealed that the instrumental learning was acquired gradually as training progressed (Figure 2A), as supported by a significant main effect of Block (*F* = 23.14, df = 5, *p* < 0.001). On the final block of training, performance was above chance level (50%) for all three associations.

A significant main effect of Group (*F* = 7.22, df = 1, *p* = 0.011) demonstrated that relative to HC, the discriminative performance was generally affected overall in PD patients (66.37 ± 16.27% vs. 78.47 ± 11.56%). We also found a significant main effect of Category (*F* = 13.39, df = 2, *p* < 0.001). Pairwise comparisons revealed that both congruent and standard performances were significantly better than incongruent performance (*p*s < 0.001), and standard performance was better than congruent performance (*p* = 0.023). We didn’t find any evidence for significant interaction effects, including the GroupCategory interaction, indicating that the group difference did not differ among the three association categories. This result was also confirmed by *post hoc* analyses (Figure 2B) that demonstrated PD patients performed worse than HC not only on the standard and congruent associations (which are predominated by the goal-directed action), but also on the incongruent association (which is predominated by the habitual control). These findings indicated that the formation of both goal-directed action and habitual control was impaired in PD patients.

In the three-way repeated-measures ANOVA of the Reaction Time (ms), we only found significant main effects of Block (*F* = 13.97, df = 5, *p* < 0.001) and Category (*F* = 15.28, df = 2, *p* < 0.001). All subjects gradually made responses faster (Figure 2C). *Post hoc* analyses demonstrated subjects responded considerably faster on both standard and congruent associations compared to the incongruent association (Figure 2D, *p*s < 0.001). These findings indicated that no trade-off effect was observed between accuracy and reaction time during the training phase. Additionally, we conducted a Chi-square test on the omission errors (time-out trials) to assess the group differences between PD and HC. Results showed that PD group had more omission errors than HC (4.75 vs. 2.98%, *p* = 0.020).

### Slips-of-Action Test

**FIGURE 3:**
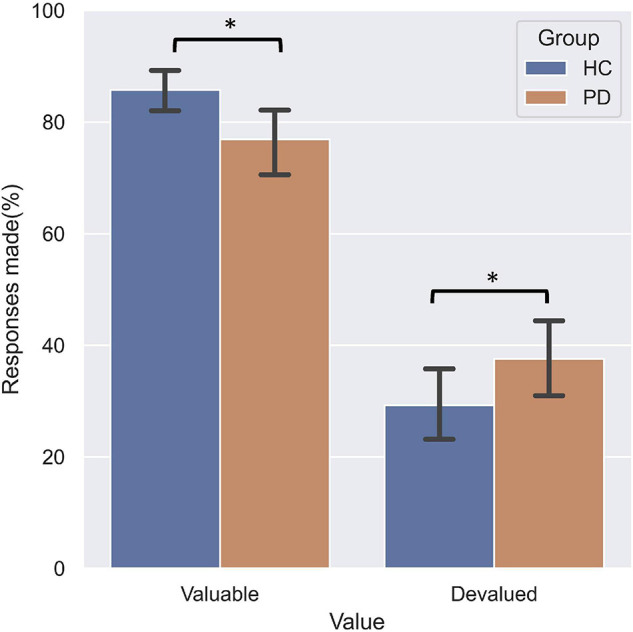
Performance on the slips-of-action test. PD patients responded significantly less for responses associated with valuable outcomes relative to HC, and more often for outcomes that were devalued.

The three-way repeated measures ANOVA on the responses made by the participants found a significant main effect of Value (*F* = 156.25, df = 1, *p* < 0.001), indicating a pronounced devaluation effect. A significant Value Category interaction effect (*F* = 51.81, df = 2, *p* < 0.001) was found. *Post hoc* analyses found that fewer responses were made for valuable outcomes and more responses for devalued outcomes on the incongruent association compared to both standard and congruent associations. Importantly, this analysis revealed a significant Group Value interaction (*F* = 4.99, df = 1, *p* = 0.031). Relative to HC (Figure 3), PD patients not only responded less for valuable outcomes (*p* = 0.035), but also responded more often to stimuli that were associated with devalued outcomes (*p* = 0.048). These findings suggest PD patients failed to engage the goal-directed system which mediated slips of action.

**FIGURE 4:**
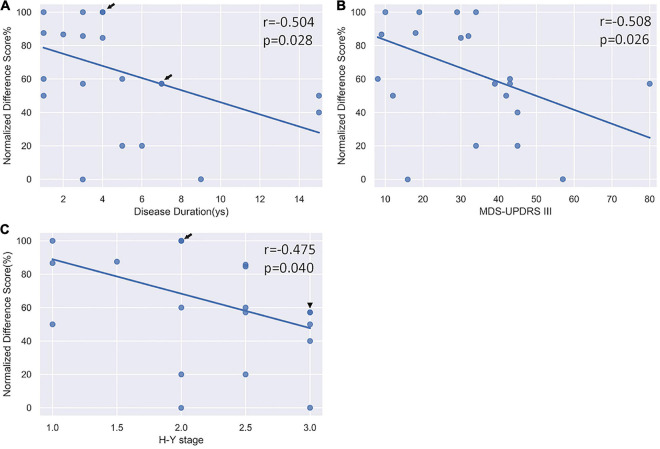
Correlation analysis between the normalized differential score and clinical severity. **(A)** A significant negative correlation with disease duration. **(B)** A significant negative correlation with MDS-UPDRS III scores. **(C)** A significant negative correlation with H-Y stage. Arrow indicates there are two subjects sharing the same value; triangle indicates there are three subjects sharing the same value.

The two-way ANOVA on the normalized difference scores revealed a main effect of Group (*F* = 4.21, df = 1, *p* = 0.047) and a main effect of Category (*F* = 28.46, df = 2, *p* < 0.001). *Post hoc* analyses showed that the normalized differential score in the PD group was significantly lower than HC (*p* < 0.001); and standard performance was significantly better than both congruent and incongruent performance (*p*s < 0.001), while the congruent was better than incongruent performance (*p* < 0.001). To investigate whether selective response suppression was related to symptom severity in the PD group, we thus further conducted Pearson correlational analyses on the normalized differential scores of the standard association (de Wit et al., 2012) with symptom severity, including disease duration, MDS-UPDRS III score, and H-Y stage. As shown in Figure 4, there were significant negative correlations between the normalized difference score and disease duration (*r* = −0.504, *p* = 0.028), MDS-UPDRS III (*r* = −0.508, *p* = 0.026), and H-Y stage (*r* = −0.475, *p* = 0.040).

### Baseline Test

**FIGURE 5:**
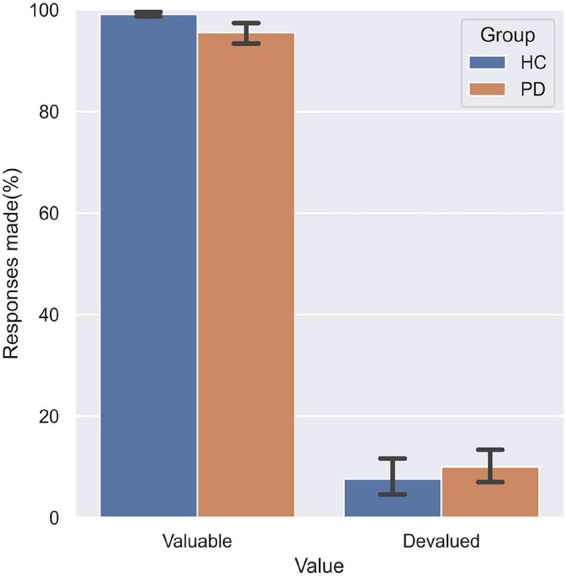
Performance on the baseline test. No significant difference was found between the two groups in the responses made to valuable and devalued outcomes.

The three-way ANOVA only revealed a significant main effect of Value (*F* = 1863.16, df = 5, *p* < 0.001, Figure 5). No significant GroupValue interaction effect was found, indicating that the overall impaired goal-directed/habitual control behaviors in PD patients were not attributable to impairments in inhibitory control.

### Explicit Knowledge Questionnaires

All participants successfully acquired explicit knowledge during the instrumental learning stage. There was a high level of explicit knowledge of response (ranging between 0 and 2) for both groups, with average scores of 1.73 and 1.81, respectively. Mean scores on the explicit knowledge outcome were 1.78 and 1.81 for the PD and HC groups, respectively. A three-way repeated measures ANOVA did not reveal any significant effect, indicating that both PD and HC subjects were able to extract the explicit knowledge for all three associations.

## Discussion

Using an instrumental training task, the present study re-addresses the acquisition of goal-directed action and habitual control that are involved in instrumental learning in PD patients. Despite prior speculation that PD subjects have selective deficits of habitual control, we demonstrate that the formation of both habitual control behavior and goal-directed action are impaired in PD patients. Then, for the first time, we employ a subsequent slips-of-action test to provide behavioral evidence for an explicit measure of the imbalance in the expression of goal-directed and habitual control behavior previously alluded to in the PD literature. Our results show that PD affects patients’ ability to adjust their behavior to the changes in outcome value, suggesting a disruption in goal-directed action control and a relatively excessive reliance on habitual control. Furthermore, we found that symptom severity is predictive of poor performance on the slips-of-action test. Taken together, these findings suggest that PD patients show a general deficit in the formation of both goal-directed action and habitual control, and exhibit an impairment in engaging the goal-directed system with a consequent relatively excessive reliance on habitual control in the expression of acquired learning.

Early in the 1980s, Mishkin and colleagues (Mishkin and Petri, 1984) had proposed that habit formation was encoded in the striatum. Since then, a large body of evidence was spawned supporting the engagement of striatum in instrumental learning (for review, see Redgrave et al., 2010; Jahanshahi et al., 2015). Moreover, dopaminergic neurons are crucially involved in the acquisition and performance of overlearned responses (Hernandez et al., 2019), and dopaminergic projections to the dorsal striatum have been implicated in the reinforcement of habits (Wise, 2004; van Nuland et al., 2020). Given the significant loss of dopamine in the SNpc and the degeneration of the dopaminergic pathways to the striatum in PD patients, it is anticipated that instrumental learning would be intrinsically impaired by PD. Indeed, consistent with several previous studies (de Wit et al., 2011; Hadj-Bouziane et al., 2012; Hayes et al., 2015; Olson et al., 2019), our results also demonstrate that the overall instrumental learning performance of PD patients is markedly impaired relative to healthy individuals. In addition, in line with a previous study using probabilistic stimulus-response task (Shohamy et al., 2004), we also found that although learning may be slower and less generalizable compared to healthy individuals, PD patients are still capable of acquiring instrumental learning over practice (Olson et al., 2019).

The pattern of neurodegeneration in PD patients affects associated learning. PD predominantly involves dopamine neurons in the SNpc ventrolateral tier (Giguere et al., 2018) and their projections to the sensorimotor striatum (Stoessl et al., 2014). The predicted consequence of such losses would particularly disturb the habitual control behavior (Wu et al., 2015a). Indeed, many of the major clinical manifestations of PD can be understood in terms of a fundamental disorder of the mechanisms responsible for automatic habitual performance (Redgrave et al., 2010; Wu et al., 2015b), such as akinesia, bradykinesia, rigidity. Consistently, in the present study, our results also reveal that PD affects patients’ ability to form habitual control behavior. Using a similar instrumental task, de Wit et al. (2011) did not find impairment in habit formation in PD patients. Such inconsistency with our current results may be attributed to the relatively milder patients enrolled in their study (mean H-Y stage of 1.7, compared to a mean of 2.2 in the present study). Instead, de Wit et al. (2011) found a disease severity–dependent impairment in goal-directed behavior. We also found that PD patients demonstrated a deficit in the formation of goal-directed action. In fact, clinical observations suggest that PD patients have difficulties with internal generation of actions and cognitive plans, instead excessively relying on cue- and stimulus-driven behavior, more in line with goal-directed impairment.

We suggest that two potential factors may contribute to the disrupted goal-directed control observed in the present study. Although not normally considered typical in PD, early significant caudate dopaminergic denervation was found in half of the cases in the Parkinson’s Progression Markers Initiative series (Pasquini et al., 2019). The caudate dopaminergic dysfunction that occurred in the early stages of the disease may therefore lead to the deficit in goal-directed actions. On the other hand, Brovelli and colleagues (Brovelli et al., 2011) have suggested that, contrary to current models, the putamen is recruited during initial acquisition and that the dynamic interplay between caudate nucleus and putamen (rather than their serial recruitment) underlies the acquisition and early consolidation of instrumental behaviors. The disrupted functional heterogeneity within the dorsal striatum may also cause impairment in goal-directed actions.

In the slips-of-action test, we found that PD patients failed to engage the goal-directed system and had an excessive reliance on habitual control relative to HC during the expression of acquired learning. Moreover, the performance on the slips-of-action was negatively correlated with metrics of disease severity, as indicated by the MDS-UPDRS III score, H-Y stage, and disease duration. The results of the baseline test suggest that the overall impaired goal-directed/habitual control behaviors in PD patients were not attributable to impairments in inhibitory control. Although there is accumulating evidence showing that habitual control activates the population of dopamine neurons that are differentially vulnerable to neurodegeneration in PD, formal investigations are still required to establish a causal association (Hernandez et al., 2019). That is, does the dopaminergic neurodegeneration cause the impairments in habitual control, or contrarily, the overuse of habitual control causes the neurodegeneration in dopamine neurons? Our findings of the relatively excessive reliance on habitual control in PD patients are actually in line with the idea proposed by Hernandez and colleagues (Hernandez et al., 2019), that the preferential depletion of dopaminergic neurotransmission in the sensorimotor striatum in PD could be explained by the critical functional stressor caused by the frequent reliance on habitual performance, when combined with other more general risk factors. Specifically, the activation of the sensorimotor striatum incurring during the performance of habitual tasks could impose an additional, potentially toxic metabolic load on the dopamine neurons that innervate this region (Hernandez et al., 2015), which would exceed a toxic threshold for dopaminergic neurons and thus cause the neuron loss. Our results show that in the expression of instrumental performance, the relatively excessive reliance on habitual control may support the theory that the habitual engagement may be a critical causal factor for the differential vulnerability of dopaminergic synapse terminals in the caudal putamen in PD.

Our study has a few limitations. First, the present study was only conducted in one medication state (OFF), thus we were not able to investigate the medication effects. In PD patients, dopaminergic therapy has been shown to redress motor and some cognitive symptoms caused by dopamine depletion primarily in the dorsal striatum; whereas appears to worsen those cognitive functions, particularly learning functions (Feigin et al., 2003; Torta et al., 2009; Jahanshahi et al., 2010; Tremblay et al., 2010), mediated by ventral tegmental area-innervated brain regions where are far less affected, presumably due to dopamine overdose of these dopamine-replete areas (Gotham et al., 1988; Cools et al., 2001; Cools, 2006; MacDonald et al., 2011). Moreover, the findings of impaired learning after administration of levodopa or dopamine agonists in healthy individuals (Santesso et al., 2009; Schnider et al., 2010; Gallant et al., 2016; Vo et al., 2016) parallel with the dopamine overdose hypothesis. At odds with this hypothesis, however, there are reports of levodopa improving learning in healthy volunteers (Knecht et al., 2004; Floel et al., 2008; Shellshear et al., 2015). This discrepancy may be explained by the fact that learning paradigms often confound learning and response-selection decisions, which are differentially affected by dopaminergic therapy (Vo et al., 2017). A previous fMRI experiment (Hiebert et al., 2014) has shown that learning stimulus-response associations from feedback correlates with preferential BOLD signal in the ventral striatum; in contrast, response selections were associated with BOLD signal in the dorsal striatum. Recently, Vo et al. (2017) reported that a single and first dose of levodopa unambiguously impaired the acquisition of stimulus-response associations in healthy young controls, whereas the experimental groups treated with levodopa or placebo did not differ in their ability to enact stimulus-specific selections once they were learned. Their results are only partially supportive of the dopamine overdose hypothesis in that stimulus-response association learning was impaired by levodopa; nevertheless, stimulus-specific response selection was not clearly affected (Vo et al., 2017). In combination with these inconsistent results reported in previous literatures, future studies with more careful design are warranted to clarify the levodopa effects on learning in PD patients. In addition, one recent study (De Houwer et al., 2018) proposed that the habitual effect in human revealed by devaluing the outcome images may have been overestimated. Indeed, there might be some other reasons that would cause a stronger slip-of-action effect, including the lack of understanding of devaluation procedure, general inhibitory deficit and cognitive impairment. However, in the present study, we matched the PD and HC subjects well on the cognitive function at the recruitment, ruled out the possibility of general inhibitory deficit using a baseline control test, and excluded participants who were not able to understand the task well. Though not all possible reasons were entirely ruled out in the present study, we suggest that the lack of goal-directed control to devalued outcomes could be used as evidence of relatively habitual control.

In summary, in the present study, we first investigated in PD patients and HC the ability to form habits by testing trial-and-error learning of S-R mappings using an instrumental association task. Our findings emphasized the impairment in the formation of Stimulus → Response habitual associations in PD patients. In addition, we also provided evidence for the deficit in the formation of goal-directed actions. Then, we further explored the features in expressing habits by assessing the balance of goal-directed and habitual control actions using a subsequent slips-of-action test. The impaired performance with progressive disease severity suggests that PD patients have difficulties in engaging the goal-directed system and present an overreliance on habitual control in the expression of habits. The present study provides further insights into understanding the effects of PD on goal-directed versus habitual behavior. We note that, however, further investigations to determine the underlying mechanisms for the instrumental dysfunction in PD are warranted. This study is carefully designed to not only address the formation process of instrumental learning, but also assess the expression process in PD patients.

## Data Availability Statement

The raw data supporting the conclusions of this article will be made available by the authors, without undue reservation.

## Ethics Statement

The studies involving human participants were reviewed and approved by the Institutional Review Board of Xuanwu Hospital of Capital Medical University. The patients/participants provided their written informed consent to participate in this study.

## Author Contributions

PC and T-MM designed the study. T-MM and WZ carried out data collection, analyzed the data, and drafted the manuscript. MM and PC revised the manuscript. All authors read and approved the final version for publication.

## Conflict of Interest

The authors declare that the research was conducted in the absence of any commercial or financial relationships that could be construed as a potential conflict of interest.

## Publisher’s Note

All claims expressed in this article are solely those of the authors and do not necessarily represent those of their affiliated organizations, or those of the publisher, the editors and the reviewers. Any product that may be evaluated in this article, or claim that may be made by its manufacturer, is not guaranteed or endorsed by the publisher.
